# Identification of gingerenone A as a novel senolytic compound

**DOI:** 10.1371/journal.pone.0266135

**Published:** 2022-03-29

**Authors:** Ruin Moaddel, Martina Rossi, Stephanie Rodriguez, Rachel Munk, Mohammed Khadeer, Kotb Abdelmohsen, Myriam Gorospe, Luigi Ferrucci

**Affiliations:** 1 Laboratory of Clinical Investigation, National Institute on Aging, Intramural Research Program, NIH, Baltimore, MD, United States of America; 2 Laboratory of Genetics and Genomics, National Institute on Aging, Intramural Research Program, NIH, Baltimore, MD, United States of America; 3 Translational Gerontology Branch, National Institute on Aging, Intramural Research Program, NIH, Baltimore, MD, United States of America; Monash University Malaysia, MALAYSIA

## Abstract

Senescent cells accumulate with aging and have been shown to contribute to age-associated diseases and organ dysfunction. Eliminating senescent cells with senolytic drugs has been shown to improve age phenotypes in mouse models and there is some initial evidence that it may improve the health of persons with chronic diseases. In this study, we employed WI-38 human fibroblasts rendered senescent by exposure to ionizing radiation (IR) to screen several plant extracts for their potential senolytic and/or senomorphic activity. Of these, ginger extract (*Zingiber officinale Rosc*.) selectively caused the death of senescent cells without affecting proliferating cells. Among the major individual components of ginger extract, gingerenone A and 6-shogaol showed promising senolytic properties, with gingerenone A selectively eliminating senescent cells. Similar to the senolytic cocktail dasatinib and quercetin (D+Q), gingerenone A and 6-shogaol elicited an apoptotic program. Additionally, both D+Q and gingerenone A had a pronounced effect on suppressing the senescence-associated secretory phenotype (SASP). Gingerenone A selectively promotes the death of senescent cells with no effect on non-senescent cells and these characteristics strongly support the idea that this natural compound may have therapeutic benefit in diseases characterized by senescent cell accumulation.

## Introduction

Senescent cells display irreversible replication arrest, as first described by Hayflick [1]. These cells often show increased activity of the senescence-associated β-galactosidase (SA-βGal) at pH 6, and increased expression of cell cycle inhibitors such as p53, CDKN1A (p21) and CDKN2A (p16) [2]. Despite their enduring growth arrest, they are metabolically active and display enhanced lysosomal activity. They also secrete a range of biologically active molecules, including pro-inflammatory cytokines, growth factors, and matrix metalloproteases, a trait known as the senescence-associated secretory phenotype (SASP) [3]. Senescence is a pleiotropic phenotype that can be beneficial in young age and detrimental in older age. For example, in young organisms, senescence was found to play a beneficial role in wound healing, tissue remodeling, embryonic development, and tumor suppression [4]. However, accumulation of senescent cells in aged tissues was found to promote premature aging and age-associated diseases such as chronic inflammation, liver fibrosis, atherosclerosis, insulin resistance, neurodegenerative disorders, and cancer [5].

In animal models, elimination of senescent cells by senolytic drugs or genetic approaches improves health span and slows down the development of aging phenotypes that are associated with functional impairment [6]. For example, the clearance of p16-positive senescent cells in the transgenic INK-ATTAC mouse improved age-associated pathologies such as atherosclerosis, neurodegeneration, sarcopenia, cataracts, cardiac hypertrophy, kidney disease, cancer, and osteoarthritis [7, 8]. Thus, the clearance of senescent cells by senotherapeutics, provides a promising avenue to combat age-related accumulation of pathological condition and functional impairments by eliminating senescence-associated traits [9]. Senotherapeutics include senolytic drugs, which selectively eliminate senescent cells by apoptosis, and senomorphics, which can either suppress the proinflammatory SASP or suppress senescent phenotypes without cell death. To-date, very few senotherapeutic compounds have been identified and validated, including navitoclax [10], fisetin, piperlongumine [11], and the senolytic cocktail dasatinib and quercetin (D+Q) [12]. More recently, extracts from plants such as *Solidago alpestris* [13] and *Deschampsia antartica* [14] were identified that displayed senolytic activity. However, the active components in these plant extracts have not been isolated or characterized.

We have previously shown that screening plant extracts is a viable approach for the identification of bio-active compounds with potential therapeutic properties [15, 16]. Thus, we hypothesized that plant extracts with known anti-inflammatory effects, may contain active compounds with senolytic and/or senomorphic properties. To this end, we screened extracts from four plants with well-established anti-inflammatory properties [17, 18], including extracts from *Harpagophytum procumbens* (Devil’s Claw), *Uncaria tomentosa* (Cat’s Claw) [19], *Zingiber officinale Rosc*. (ginger) [20] and *Solidago canadensis* (Canadian Goldenrod). The extract from *Zingiber officinale Rosc*. (ginger) was found to reduce both senescent cell viability and SA-βGal activity. Gingerenone A was the most promising component identified in ginger extract, since it reduced senescent cell viability, enhanced cleaved caspase-3, decreased levels of the anti-apoptotic protein Bcl-XL, and decreased production of the SASP proinflammatory cytokine IL-6. Together, our findings suggest that gingerenone A suppresses several senescence traits and promotes senolysis, thus representing a promising natural senolytic compound.

## Methods

### Materials

Dulbecco’s modified Eagle’s medium, 10% heat inactivated fetal bovine serum, penicillin and streptomycin and non-essential amino acids were purchased from Gibco (ThermoFisher Scientific) and RIPA lysis buffer was purchased from Pierce (ThermoFisher Scientific, Waltham, Massachusetts). Protease and phosphatase inhibitors were purchased from Roche (ThermoFisher Scientific). Gingerenone A was purchased from Aobious (Gloucester, MA) and 6-shogaol, dasatinib and zingerone were purchased from Cayman Chemical (Ann Arbor, MI). 6-gingerol, 8-gingerol 10-gingerol, 8-shogaol and quercetin were purchased from Sigma-Aldrich (Milwaukee, WI). Cell counting was performed by using a TC20 Automated Cell Counter (BioRad, Hercules, CA). Pictures were acquired by using a digital camera system (Nikon Digital Sight) for microscope (Nikon Eclipse S100) (Nikon Instruments, Melville, NY). 24-well plates and 6-well plates were purchased from Fisher Scientific. *Harpagophytum procumbens* (Devil’s Claw) (herb to menstruum ratio, 1:3), *Uncaria tomentosa* (Cat’s Claw) (herb to menstruum ratio, 1:3), *Zingiber officinale Rosc*. (ginger) (herb to menstruum ratio, 1:3) and *Solidago canadensis* (Canadian Goldenrod) (herb to menstruum ratio, 1:3) were purchased from Galen’s Way (Sebastopol, CA). The MTT assay was purchased from BioVision (Milpitas, CA). Senescence β-Galactosidase Staining Kit, ID # 9860S was purchased from Cell Signaling Technology (Danvers, MA). Primary antibodies recognizing p21, p53, Beta-Actin (ACTB) were purchased from Santa Cruz Biotechnology (Dallas, Texas), primary antibodies recognizing caspase-3 were purchased from Abcam (Cambridge, MA) and Bcl-XL from Cell Signaling (Danvers, MA). IL-6 expression was measured by the Quantikine ELISA Kit purchased from R&D systems (Minneapolis, MN). Cytokines and chemokines were measured by multiplex assays including customized Luminex plates purchased from R &D (Minneapolis, MN) and V-Plex plus Proinflammatory panel 1 Human kit and the Chemokine Panel 1 Kit were purchased from Mesoscale Diagnostics (Rockville, MD).

### Cell culture and treatments

WI-38 human diploid fibroblasts (HDFs) were cultured in Dulbecco’s modified Eagle’s medium (DMEM) supplemented with 10% heat-inactivated fetal bovine serum (FBS), penicillin and streptomycin, and non-essential amino acids at 37°C in a 5% CO_2_ incubator. HDFs were rendered senescent by exposure to 10 Gy of ionizing radiation (IR) and then cultured for an additional ten days. Proliferating and senescent WI-38 cells were seeded in triplicate overnight at 2×10^5^ cells/well (in 6 well plates) to achieve ∼60% confluence by the next day. Cells were then treated with the extracts for times indicated.

In 24-well plates, cells were treated with multiple dilutions (1:200 and 1:500) of *Harpagophytum procumbens* (Devil’s Claw) (herb to menstruum ratio, 1:3), *Uncaria tomentosa* (Cat’s Claw) (herb to menstruum ratio, 1:3), *Zingiber officinale Rosc*. (ginger) (herb to menstruum ratio, 1:3) and *Solidago canadensis* (Canadian Goldenrod) (herb to menstruum ratio, 1:3) (Galen’s Way, Sebastopol, CA). In 6-well plates, cells were treated for 24 or 48 h with the corresponding amount of DMSO (0.1%), D+Q (250 nM dasatinib and 10 μM quercetin), gingerenone A (20 μM) and 6-shogaol (72.4 nM). Cell counting was performed by using a TC20 Automated Cell Counter (BioRad). Pictures were acquired by using a digital camera system (Nikon Digital Sight) for microscope (Nikon Eclipse TS100).

### MTT cell viability

The MTT assay (BioVision Milpitas, CA) was carried out following the manufacturer’s protocol. Briefly, the media was gently removed from each well and 50 μl of serum-free medium + 50 μl of Solution A were added to each well. The plates were incubated at 37°C for MTT reduction into formazan for 3 h. Next, 150 μl Solution B was added to each well and the plate was covered with foil and shaken at 600 rpm for 15 min. The absorbance was measured at 590 nm using Enspire, Perkin Elmer Multimode plate reader.

### SA-βGal assay

Senescence-associated β-galactosidase (SA-βGal) activity in WI-38 cells was assessed by the Senescence β-Galactosidase Staining Kit following the manufacturer’s protocol (Cell Signaling Technology). Briefly, the growth medium was aspirated from the 24-well plates, the wells were washed once with 0.5 ml of 1xPBS, 250 μl of Fixative solution (1X) was added to each well, and the plate was incubated at room temperature for 15 min. The solution was removed, the wells were washed twice with 0.5 ml of 1x PBS, and 250 μl of 1X SA-βGal detection solution was added. The plate was sealed with parafilm to prevent evaporation and was incubated overnight at 37°C with no CO_2_. The SA-βGal detection solution was removed, 250 μl of 1x PBS was added, and the plate was gently shaken for 1 h at room temperature. The PBS was removed, and 70% glycerol was added to each well for long term storage.

### Protein analysis

Cells were lysed in RIPA lysis buffer (Pierce, ThermoFisher) containing protease and phosphatase inhibitors (Roche) and incubated on ice for 10 min. The lysates were then sonicated for 5 min and centrifuged for 10 min at 4°C to remove the insoluble fraction. The remaining supernatant was the whole-cell lysate. Lysates were separated by SDS-polyacrylamide gel electrophoresis (SDS-PAGE) and transferred onto nitrocellulose membranes (BioRad). Incubations with primary antibodies recognizing p21, p53, Beta-Actin (ACTB) (Santa Cruz Biotechnology), as well as caspase-3 (Abcam) and Bcl-XL (Cell Signaling) were followed by incubations with the appropriate secondary antibodies conjugated with horseradish peroxidase (GE Healthcare). Signals were developed using Enhanced Chemiluminescence (ECL) and acquired by using the ChemiDoc MP Imaging System (BioRad).

### Secreted protein analysis: ELISA, Luminex Multiplex, and V-PLEX

Media was collected at the earliest timepoint (24 h after treatments) to avoid cell death that could interfere with the assay. The media was centrifuged at 1000 x g for 5 min to remove debris. The secretion of IL-6 was measured by the Quantikine ELISA Kit (R&D systems) and the expression of pro-inflammatory cytokines and chemokines [IL-6, TNFα, CCL2 (MCP-1), IL-8, IFNβ] was also assessed by multiplex assays (customized Luminex plate, R&D) according to the manufacturer’s instructions. The V-PLEX Plus Proinflammatory Panel 1 Human Kit and the Chemokine Panel 1 preparation and detection were carried out following the manufacturer’s protocol (Mesoscale Diagnostics) using >80% confluency of proliferating or senescent cells on 24-well plates. Briefly, different concentrations of standards and samples were added to the plates, and they were incubated for 2 h on a microplate shaker. The plates were washed, and the corresponding detection antibody mixture was added to each well and incubated for 1 h with shaking. The plates were washed twice then Read buffer was added to each well. The plates were read on the MESO Quickplex SQ 120 (MSD, Rockville, MD) and protein concentrations were determined using MSD Discovery workbench 4.0.

## Results

### Screening identifies senolytic properties in ginger extract

**Fig 1:**
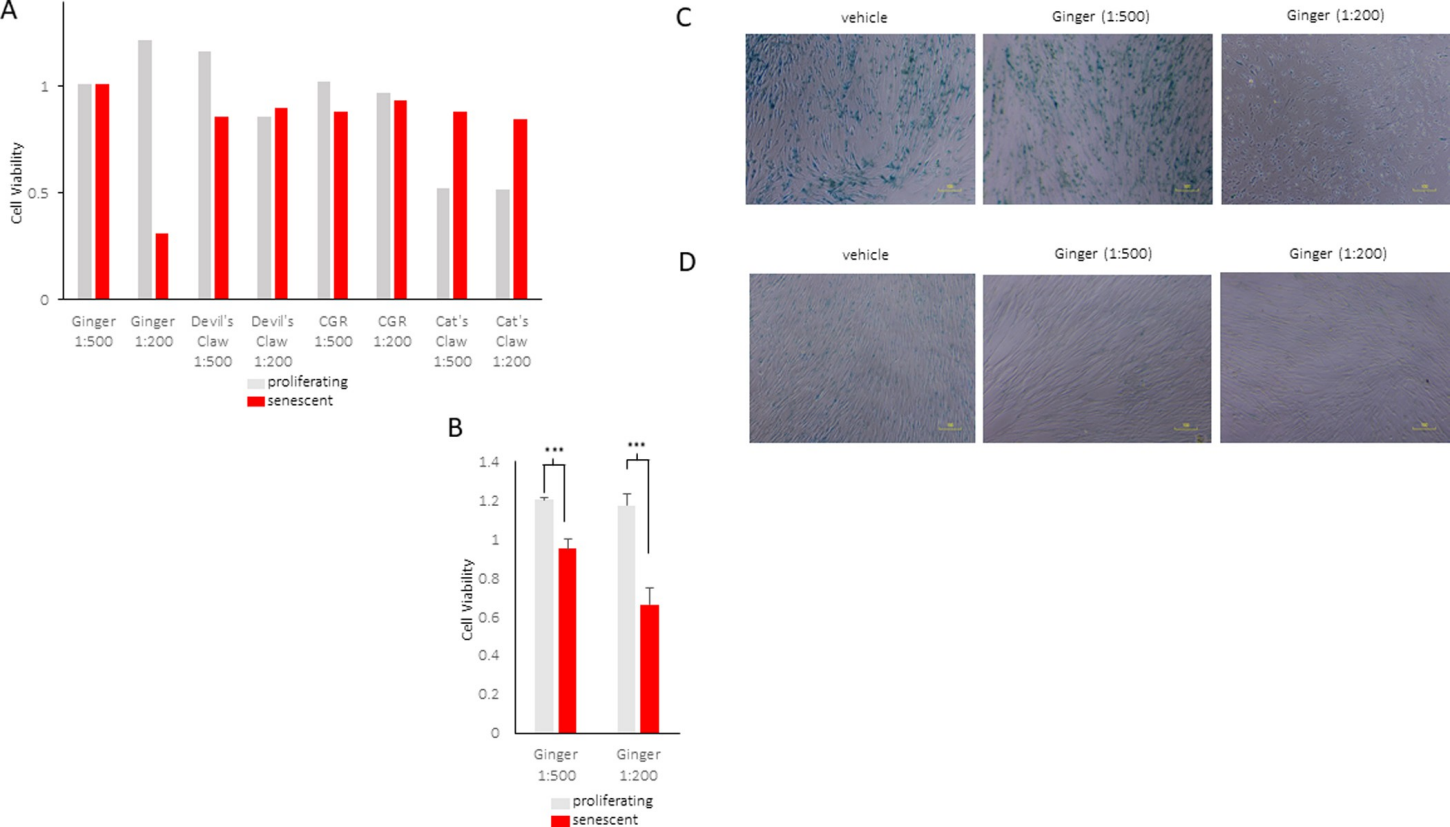
Extract screening reveals senolytic properties of ginger. (A) WI-38 fibroblasts were left untreated (grey) or rendered senescent by exposure to ionizing radiation (IR, 10 Gy) followed by culture for an additional 10 days (red), whereupon cells were treated with the indicated extracts and dilutions for 24 h. (B) The impact on ginger extract on proliferating (grey) and senescent (red) cells on cell survival, assessed by the MTT cell viability assay. Data are represented as the mean and standard errors from biological replicates [senescent n = 9 and proliferating (1:500 (n = 4) and 1:200 (n = 7), (***p<0.005)]. (C) Cells that were prepared as in (A) were treated with ginger extract for 24 h or (D) 72 h followed by senescence-associated β-galactosidase (SA-βGal) staining.

Four plant extracts were screened for senolytic activity, including extracts from *Harpagophytum procumbens* (Devil’s Claw), *Uncaria tomentosa* (Cat’s Claw), *Zingiber officinale Rosc*. (ginger) and *Solidago canadensis* (Canadian Goldenrod). These extracts were screened using the MTT cell viability assay. To avoid nonspecific effects, proliferating cells and cells rendered senescent by exposure to ionizing radiation (IR) were used as a screening system. IR senescence was triggered by exposure to 10 Gy followed by culture for an additional 10 days. As shown in (Fig 1A and 1B), only ginger extract (1:200 dilution) markedly decreased senescent cell viability compared to other extracts. Interestingly, incubation with extracts from *Uncaria tomentosa* decreased cell viability in proliferating cells but had no effects on IR-induced senescent cells (Fig 1A). Incubation with ginger extracts displayed senolytic activity (Fig 1B) and decreased SA-βGal activity at 24 and 72 h (Fig 1C and 1D, respectively), suggesting a possible shift in senescence traits. While this effect could be due to a reduction in the number of senescent cells, it could also result from the inhibition of SA-βGal activity by ginger extracts.

### Gingerenone A induces senescent cell death

**Fig 2:**
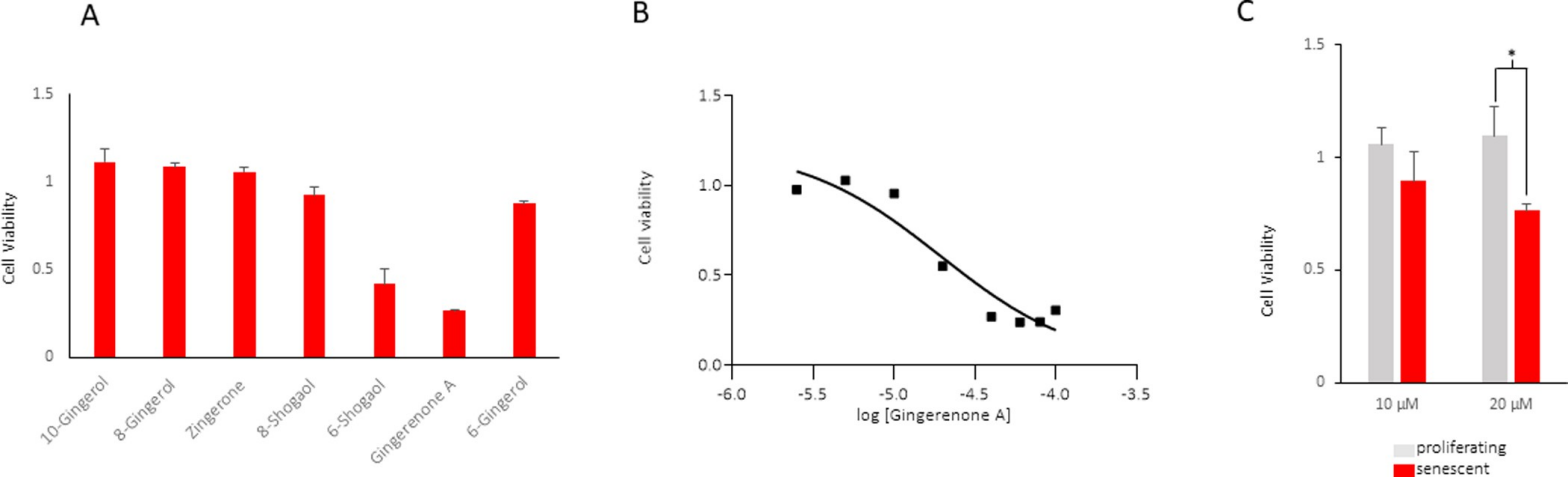
Senolytic activity of gingerenone A. (A) WI-38 fibroblasts were rendered senescent by exposure to ionizing radiation (IR, 10 Gy) and cultured for an additional 10 days. Cells were then treated with the ginger components at 100 μM, with 6-shogaol at 3.6 μM, 8-shogaol at 33 μM and 6-gingerol at 20 μM for 72 h, and then cell survival was assessed by the MTT cell viability assay. Data represent the means and standard error from three biological replicates. (B) Dose-response effect of gingerenone A [2.5–100 μM] treatments for 72 h on senescent cell viability 10-days post-irradiation. (C) Gingerenone A was incubated with cells that were either proliferating (grey) or senescent (red) as explained in panel (A) for 48 h at 10 and 20 μM (*p = 0.04). Data represent the means and standard error from eight biological replicates.

We used a candidate approach to identify the active senolytic component within the ginger extract. The major components of ginger extract, namely 6-gingerol, 8-gingerol, 10-gingerol, 6-shogaol, 8-shogaol, gingerenone A, and zingerone, were screened for their ability to selectively decrease cell viability of senescent cells using the MTT assay (Fig 2A). Among the active components, only 6-shogaol and gingerenone A displayed senolytic activity. The IC_50_ of gingerenone A was determined to be 19.6 ± 2.1 μM (r^2^ = 0.9214) (Fig 2B). Treatment with 20 μM gingerenone A resulted in a significant decrease in senescent cell viability relative to proliferating cells (p = 0.04) (Fig 2C).

**Fig 3:**
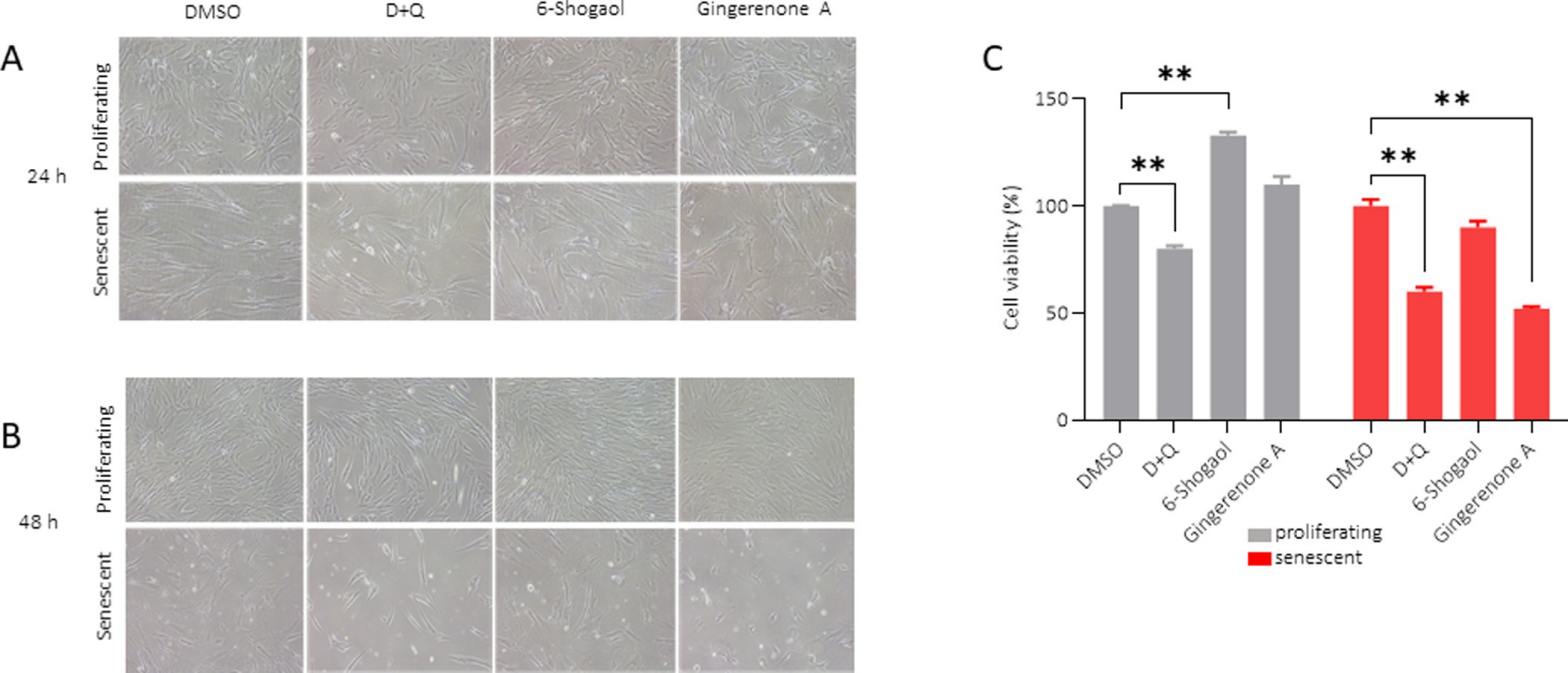
Gingerenone A and 6-shogaol have senolytic effects. WI-38 fibroblasts were either left untreated (proliferating) or rendered senescent by exposure to ionizing radiation (IR, 10 Gy) and cultured for an additional 10 days. Cells were then treated with either vehicle (DMSO), dasatinib (250 nM) and quercetin (10 μM) (D+Q), gingerenone A (20 μM), or 6-shogaol (72.4 nM) and incubated for 24 h (A) or 48 h (B), whereupon cell viability was assayed by MTT analysis [proliferating (grey) and senescent (red)]. (C) Data in graphs represent the means and standard error from three biological replicates. Micrographs are representative of three biological replicates.

The cytotoxic action of gingerenone A was compared with that of the known senolytic D+Q. Senescent cells were treated with either 20 μM gingerenone A,72.4 nM of 6-shogaol or D+Q for 24 h and 48 h (Fig 3A and 3B, respectively). Forty-eight hours after treatment, D+Q showed a decrease in senescent cell viability, with a moderate toxic effect on proliferating cells (Fig 3B and 3C). Interestingly, treatment with 72.4 nM of 6-shogaol induced a significant increase in the number of proliferating cells (Fig 3C) and showed a moderate degree of cell death in senescent cells. Treatment with gingerenone A did not show any significant effect on the proliferation of non-irradiated cells, however it significantly decreased senescent cell viability, and showed a higher selectivity compared to D+Q.

### Gingerenone A suppresses the SASP

**Fig 4:**
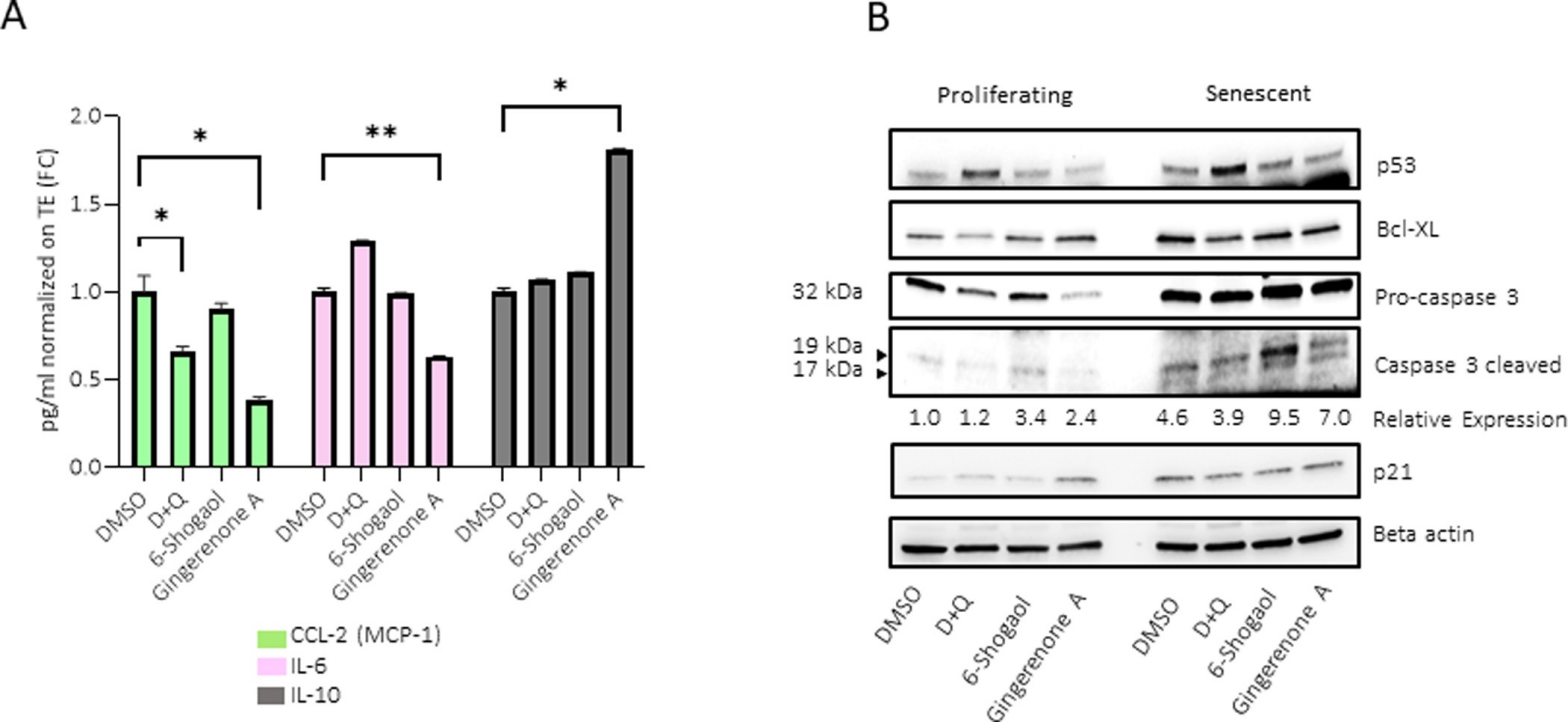
Gingerenone A suppresses SASP and enhances apoptosis in senescent cells. (A) WI-38 fibroblasts were rendered senescent by exposure to ionizing radiation (IR, 10 Gy) and cultured for an additional 10 days. Cells were then treated with gingerenone A (20 μM) and 6-shogaol (72.4 nM) and incubated for 24 h. CCL-2 (MCP-1) (green) and IL-10 (grey) were measured using the Bio-plex (Multiplex); IL6 (pink) was measured using Quantikine IL-6 (R&D). Data were normalized to cell number/total protein content. Data in graphs represent the means and standard error from three biological replicates. (B) Western blot analysis of the proteins shown after treating proliferating and senescent fibroblasts (as explained in panel A) for 48 h with the drugs indicated. Ratios of cleaved pro-caspase 3 to total caspase 3, normalized to ACTB levels, shown as relative to control proliferating cells.

The effects of gingerenone A, 6-shogaol and D+Q on cytokines and chemokines that are known to be part of the SASP were tested (Fig 4, S1 Fig). Treatment with gingerenone A reduced the secreted levels of the pro-inflammatory factors IL-6, CCL2 (MCP-1) (Fig 4A) and interferon γ-induced protein 10 (IP-10) (S1B Fig) and increased the levels of the anti-inflammatory cytokines IL-10 and IL-13 (Fig 4A and S1A Fig). However, treatment with gingerenone A also increased the levels of pro-inflammatory cytokines IL-8 and IL-1β (S1A Fig). While treatment with D+Q resulted in a similar reduction in secreted MCP-1 and IP-10 levels, it also reduced the levels of IL-10 and IL-8 (S1A Fig), with enhanced secretion of the pro-inflammatory cytokines IL-6, IL-4 and IFN-G (IFNγ) (S1A Fig). Conversely, treatments with 6-shogaol did not induce major changes in any of the cytokines and chemokines measured at the tested concentration, suggesting that 6-shogaol does not have senomorphic effects compared to gingerenone A.

These results suggest that the senomorphic effects of gingerenone A and D+Q occur through somewhat different mechanisms. Of note, while IL-6, IL-8 and MCP-1 (CCL2) had similar results across different assay platforms, IL-10, which showed an increase with gingerenone A when measured by multiplex ELISA (Fig 4A), was unchanged in the V-Plex (S1 Fig). Whether this discrepancy results from the different antibodies used in the different platforms remains unknown.

### Gingerenone A enhances apoptosis in senescent cells

To determine whether gingerenone A and 6-shogaol might influence the apoptotic pathway, their effect on the levels of cleaved caspase-3 (pro-apoptotic marker), and Bcl-XL (anti-apoptotic protein) (Fig 4B) was tested. Treatment with D+Q, but not with gingerenone A or 6-shogaol, increased p53 levels in both proliferating and senescent cells. However, D+Q, gingerenone A, and 6-shogaol all reduced the expression of the anti-apoptotic protein Bcl-XL in senescent cells (Fig 4B, *right*). Interestingly, both gingerenone A and 6-shogaol induced an increase in the levels of the pro-apoptotic protein cleaved caspase-3 in senescent cells. These results indicate that gingerenone A may induce senescent-cell death through caspase-3 and independently of p53.

## Discussion

Senescence is an enduring state of cell cycle arrest that occurs following exposure to different types of stresses. Senescence has anti-tumorigenic properties in young organisms by halting growth of damaged cells, but accumulation of senescent cells can be detrimental in older age as it promotes inflammation, tumorigenesis, and age-related pathologies. Senotherapies, including senolytics and/or senomorphics, attempt to selectively target senescent cells to mitigate their deleterious effects. To this end, we screened multiple extracts to identify those that cause selective reduction in senescent cells viability. As inflammation is a hallmark of senescence [21], four plant extracts with known anti-inflammatory effects were tested, including those obtained from *Harpagophytum procumbens* (Devil’s Claw), *Uncaria tomentosa* (Cat’s Claw), *Zingiber officinale Rosc*. (ginger) and *Solidago canadensis* (Canadian Goldenrod). Among the extracts tested, ginger extract displayed robust senolytic activity. Using a candidate approach, gingerenone A and 6-shogaol were identified as active components in ginger extract, with gingerenone A showing higher selectivity for eliminating senescent cells when compared to a known senolytic cocktail (D+Q).

We studied the senomorphic effects of gingerenone A and 6-shogaol and the secretion of SASP factors by senescent cells. The activation of the SASP comprises a range of chemokines, pro-inflammatory cytokines, growth factors and matrix-remodeling enzymes that affect their microenvironment [21]. The senomorphic effects of gingerenone A were demonstrated by the reduced secretion of pro-inflammatory factors, including IL-6, IP-10 and MCP-1, and the increase in anti-inflammatory cytokines IL-10 and IL-13. The increase in anti-inflammatory cytokines is consistent with a recent study in which gingerenone A prevented local inflammation by inhibiting macrophage recruitment and inflammatory cytokine expression [22]. Further, IL-13 levels were reported to decrease in premature senescence triggered by proteasome inhibition [23]. However, gingerenone A was also found to increase the secretion of potent pro-inflammatory SASP cytokines (IL-1B and IL-8) in senescent cells. The divergence of IL-6 and IL-8 secretion with gingerenone A treatment suggests that the mechanism is independent of IL-1A (IL-1α), which can control activation of IL-6 and IL-8 via amplification of C/EBPβ activation [21]. Interestingly, while 6-shogaol is known to have strong anti-inflammatory properties [24], it did not display senomorphic effects in our study. Therefore, the anti-inflammatory impacts of 6-shogaol may not translate into senomorphic effects on senescent cells. This notion was further supported in our study suggesting that the senomorphic effect of gingerenone A and D+Q occurred through different mechanisms (Fig 4).

Previous studies suggested that gingerenone A plays a role in regulating fatty acid metabolism and mitochondrial biogenesis by activating 5’ AMP-activated protein kinase (AMPK) [22]. AMPK has shown to be inactivated in senescent cells and its activation was beneficial for senescence prevention [25]. AMPK activation was also shown to reduce abnormal inflammatory responses and lung cellular senescence [26] and in a separate study restore the NAD(+) levels in the senescent cells via the salvage pathway [25]. Treatment with gingerenone A did not increase p53 levels in proliferating or senescent cells, but it reduced the expression of Bcl-XL in senescent cells, resulting in an increase in caspase-3, strongly suggesting that gingerenone A operates through caspase-3 cleavage and independently of p53 activity.

The anti-inflammatory effects of gingerenone A observed in our study are consistent with its senolytic/senormorphic effects due to the association between senescence and chronic inflammation. Our results demonstrate that gingerenone A plays a role in inflammation and immune system regulation and has been identified as a novel senolytic/senomorphic through caspase-3 cleavage. Additional investigation is needed to fully characterize the detailed mechanism of its senolytic/senomorphic mechanistic effects. This study further reveals a screening method suitable for identifying plant extracts and their active components with senolytic activity in culture. Further studies are needed to demonstrate senotherapeutic activity *in vivo*.

## Supporting information

S1 Fig The effects of gingerenone A and 6-shogaol on cytokines and chemokines secretion in senescent cells. WI-38 fibroblasts were rendered senescent by exposure to ionizing radiation (IR, 10 Gy) and cultured for an additional 10 days. Cells were then treated with gingerenone A (20 μM) and 6-shogaol (72.4 nM) and were incubated for 24 h, when cytokines (A) and chemokines (B) were measured using the V-plex panel (MSD). Relative Secretion was measured as pg/ml vs control normalized on TE. Data in graphs represent the means and standard error from three biological replicates.
(PDF)

S1 Raw images (PDF)

## References

[1] Hayflick L, Moorhead PS. The serial cultivation of human diploid cell strains. Exp. Cell Res. 1961; 25:585–621. doi: 10.1016/0014-4827(61)90192-6 13905658

[2] Kuilman T, Michaloglou C, Mooi WJ, Peeper DS. The essence of senescence. Genes & development, 2010; 24(22), 2463–2479. 10.1101/gad.1971610 21078816 PMC2975923

[3] Bhaumik D, Scott GK, Schokrpur S, Patil CK, Orjalo AV, Rodier F, et al. MicroRNAs miR-146a/b negatively modulate the senescence-associated inflammatory mediators IL-6 and IL-8. Aging (Albany NY). 2009 Apr;1(4):402–11. doi: 10.18632/aging.100042 20148189 PMC2818025

[4] Tominaga K. The emerging role of senescent cells in tissue homeostasis and pathophysiology. Pathobiol Aging Age Relat Dis. 2015;5:27743. doi: 10.3402/pba.v5.27743 25994420 PMC4439419

[5] McHugh D, Gil J. Senescence and aging: Causes, consequences, and therapeutic avenues. J Cell Biol. 2018;217(1):65–77. doi: 10.1083/jcb.201708092 29114066 PMC5748990

[6] Kirkland JL, Tchkonia T. Senolytic drugs: from discovery to translation. J Intern Med. 2020;288(5):518–536. doi: 10.1111/joim.13141 32686219 PMC7405395

[7] Baker DJ, Wijshake T, Tchkonia T, LeBrasseur NK, Childs BG, van de Sluis B, et al. Clearance of p16Ink4a-positive senescent cells delays ageing-associated disorders. Nature. 2011;479(7372):232–6. doi: 10.1038/nature10600 22048312 PMC3468323

[8] Childs BG, Gluscevic M, Baker DJ, Laberge RM, Marquess D, Dananberg J, van Deursen JM. Senescent cells: an emerging target for diseases of ageing. Nature reviews. Drug discovery. 2017; 16(10), 718–735. doi: 10.1038/nrd.2017.116 28729727 PMC5942225

[9] Tchkonia T, Zhu Y, van Deursen J, Campisi J, Kirkland JL. Cellular senescence and the senescent secretory phenotype: therapeutic opportunities. J Clin Invest. 2013;123(3):966–72. doi: 10.1172/JCI64098 23454759 PMC3582125

[10] Zhu Y, Tchkonia T, Fuhrmann-Stroissnigg H, Dai HM, Ling YY, Stout MB, et al. Identification of a novel senolytic agent, navitoclax, targeting the Bcl-2 family of anti-apoptotic factors. Aging Cell. 2016;15(3):428–35. doi: 10.1111/acel.12445 26711051 PMC4854923

[11] Zhu Y, Doornebal EJ, Pirtskhalava T, Giorgadze N, Wentworth M, Fuhrmann-Stroissnigg H, et al. New agents that target senescent cells: the flavone, fisetin, and the BCL-XL inhibitors, A1331852 and A1155463. Aging (Albany NY). 2017;9(3):955–963. doi: 10.18632/aging.101202 28273655 PMC5391241

[12] Xu M, Pirtskhalava T, Farr JN, Weigand BM, Palmer AK, Weivoda MM, et al. Senolytics improve physical function and increase lifespan in old age. Nat Med. 2018;24(8):1246–1256. doi: 10.1038/s41591-018-0092-9 29988130 PMC6082705

[13] Lämmermann I, Terlecki-Zaniewicz L, Weinmüllner R, Schosserer M, Dellago H, Dargen de Matos Branco A, et al. Blocking negative effects of senescence in human skin fibroblasts with a plant extract. NPJ Aging and Mech. Dis. 2018;4:4. doi: 10.1038/s41514-018-0023-5 29675264 PMC5895844

[14] Ortiz-Espín A, Morel E, Juarranz Á, Guerrero A, González S, Jiménez A, Sevilla F. An Extract from the Plant Deschampsia antarctica Protects Fibroblasts from Senescence Induced by Hydrogen Peroxide. Oxid Med Cell Longev. 2017; 2017:2694945. doi: 10.1155/2017/2694945 28894504 PMC5574316

[15] Yasuda M, Wilson DR, Fugmann SD, Moaddel R. Synthesis and characterization of SIRT6 protein coated magnetic beads: identification of a novel inhibitor of SIRT6 deacetylase from medicinal plant extracts. Anal Chem. 2011;83(19):7400–7. doi: 10.1021/ac201403y 21854049 PMC3197717

[16] Dossou KSS, Devkota KP, Morton C, Egan JM, Lu G, Beutler JA, Moaddel R. Identification of CB1/CB2 Ligands from Zanthoxylum bungeanum. J.Nat.Products.2013;76(11):2060–4. doi: 10.1021/np400478c 24175626 PMC8385540

[17] Beg S, Swain S, Hasan H, Abul Barkat M, Hussain S. Systematic review of herbals as potential anti-inflammatory agents: Recent advances, current clinical status and future perspectives. Pharmacogn. Rev. 2011;5(10): 120–137. doi: 10.4103/0973-7847.91102 22279370 PMC3263046

[18] Conrozier T, Mathieu P, Bonjean M, Marc JF, Renevier JL, Balblanc JC. A complex of three natural anti-inflammatory agents provides relief of osteoarthritis pain. Altern. Ther. Health Med. 2014;20, Suppl. 1: 32–7. 24473984

[19] Sandoval M, Okuhama NN, Zhang XJ, Condezo LA, Lao J, Angeles’ FM, et al. Anti-inflammatory and antioxidant activities of cat’s claw (Uncaria tomentosa and Uncaria guianensis) are independent of their alkaloid content. Phytomedicine. 2002;9(4):325–37. doi: 10.1078/0944-7113-00117 12120814

[20] Mashhadi NS, Ghiasvand R, Askari G, Hariri M, Darvishi L, Mofid MR. Anti-oxidative and anti-inflammatory effects of ginger in health and physical activity: review of current evidence. Int J Prev Med. 2013;4(Suppl 1):S36–42. 23717767 PMC3665023

[21] Lasry A, Ben-Neriah Y. Senescence-associated inflammatory responses: aging and cancer perspectives,Trends in Immunology. 2015;36(4), 217–228. doi: 10.1016/j.it.2015.02.009 25801910

[22] Suk S, Kwon GT, Lee E, Jang WJ, Yang H, Kim JH, et al. Gingerenone A, a polyphenol present in ginger, suppresses obesity and adipose tissue inflammation in high-fat diet-fed mice. Mol Nutr Food Res. 2017;61(10): doi: 10.1002/mnfr.201700139 28556482 PMC5947313

[23] Maciel-Barón LA, Morales-Rosales SL, Aquino-Cruz AA, Triana-Martínez F, Galván-Arzate S, Luna-López A, et al. Senescence associated secretory phenotype profile from primary lung mice fibroblasts depends on the senescence induction stimuli. Age (Dordr). 2016;38(1):26. doi: 10.1007/s11357-016-9886-1 26867806 PMC5005892

[24] Dugasani S, Pichika MR, Nadarajah VD, Balijepalli MK, Tandra S, Korlakunta JN. Comparative antioxidant and anti-inflammatory effects of [6]-gingerol, [8]-gingerol, [10]-gingerol and [6]-shogaol. J Ethnopharmacol. 2010;127(2):515–20. doi: 10.1016/j.jep.2009.10.004 19833188

[25] Han X, Tai H, Wang X, Wang Z, Zhou J, Wei X, et al. AMPK activation protects cells from oxidative stress-induced senescence via autophagic flux restoration and intracellular NAD(+) elevation. Aging Cell. 2016;15(3):416–27. doi: 10.1111/acel.12446 26890602 PMC4854918

[26] Cheng XY, Li YY, Huang C, Li J, Yao HW. AMP-activated protein kinase reduces inflammatory responses and cellular senescence in pulmonary emphysema. Oncotarget. 2017;8(14):22513–22523. doi: 10.18632/oncotarget.15116 28186975 PMC5410241

