# Supplementary figures and images for: Identification of gingerenone A as a novel senolytic compound
Source: PLoS One. 2022 Mar 29;17(3):e0266135. doi: 10.1371/journal.pone.0266135 (PMC8963586; doi:10.1371/journal.pone.0266135)

A

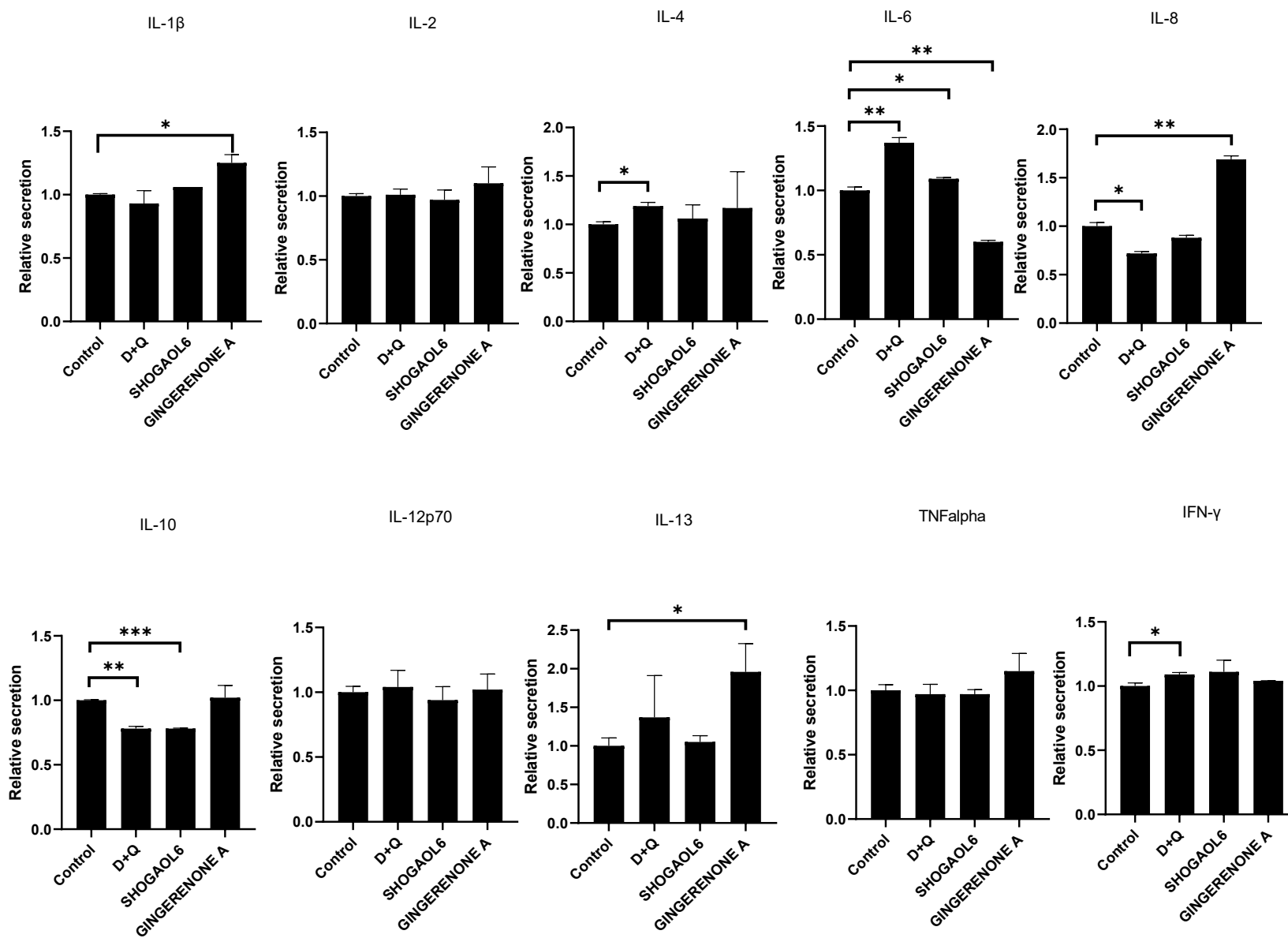

**B**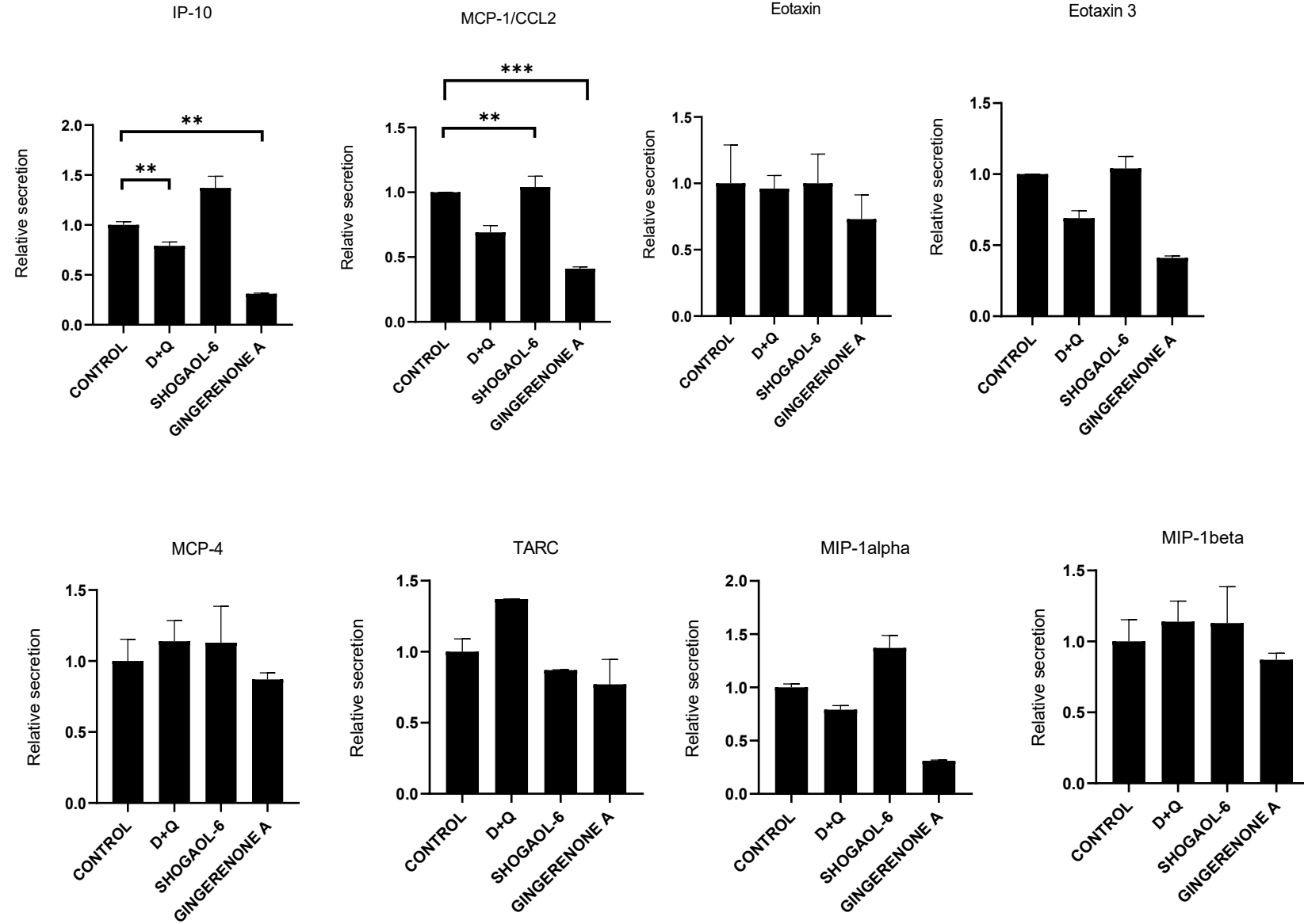

Supplement: S1 Fig — WI-38 fibroblasts were rendered senescent by exposure to ionizing radiation (IR, 10 Gy) and cultured for an additional 10 days. Cells were then treated with gingerenone A (20 μM) and 6-shogaol (72.4 nM) and were incubated for 24 h, when cytokines (A) and chemokines (B) were measured using the V-plex panel (MSD). Relative Secretion was measured as pg/ml vs control normalized on TE. Data in graphs represent the means and standard error from three biological replicates. (PDF) [file pone.0266135.s001.pdf]

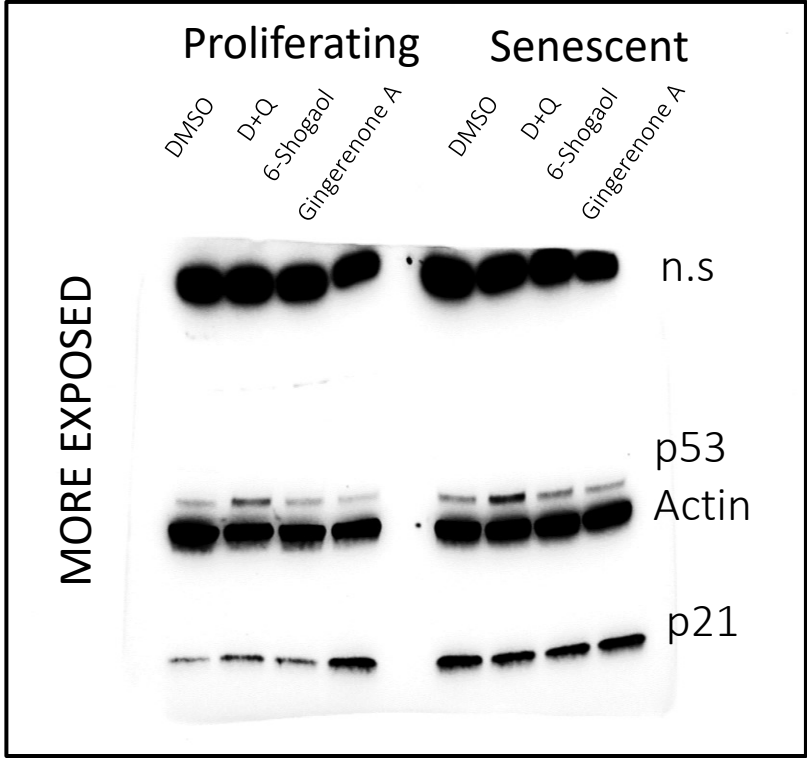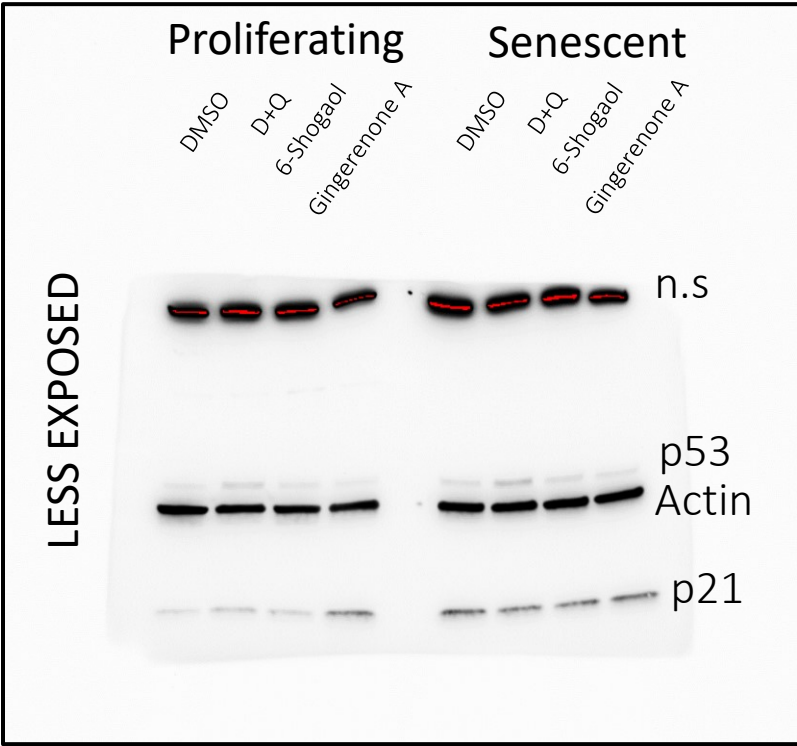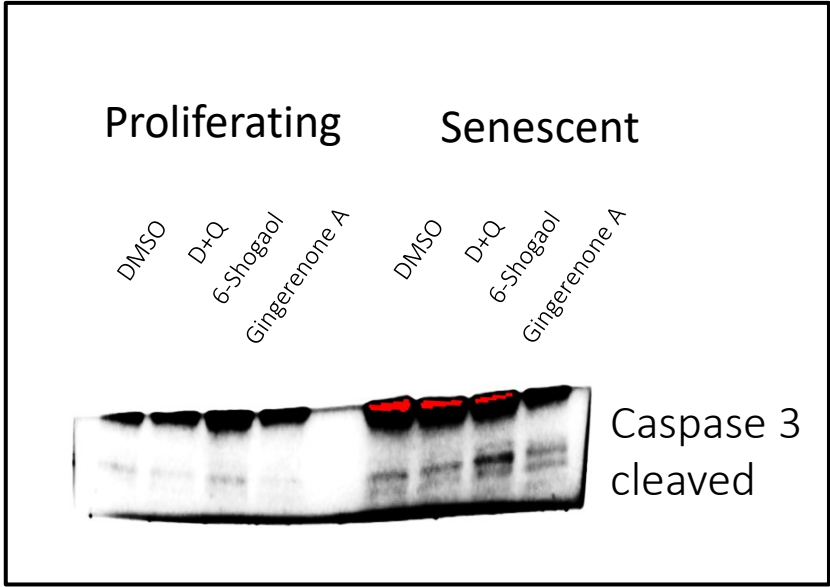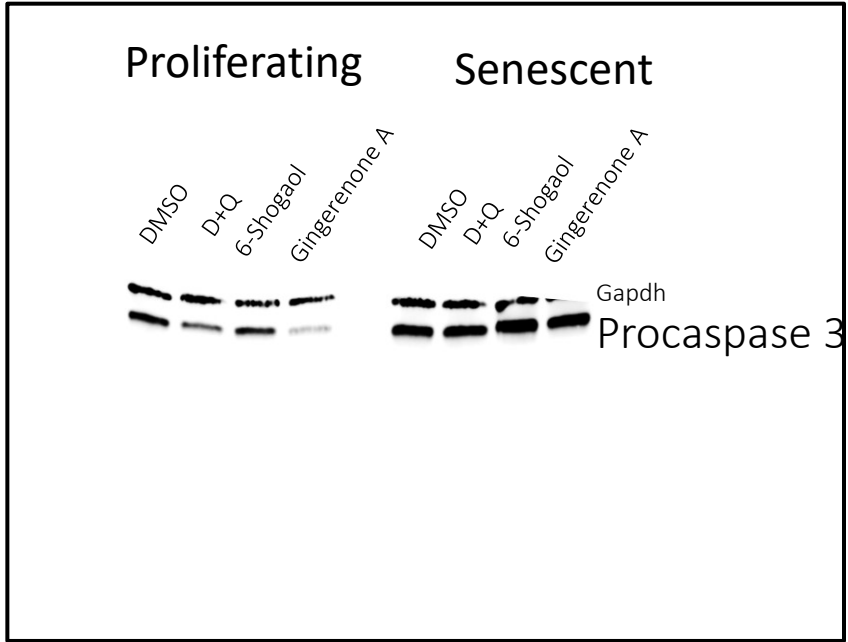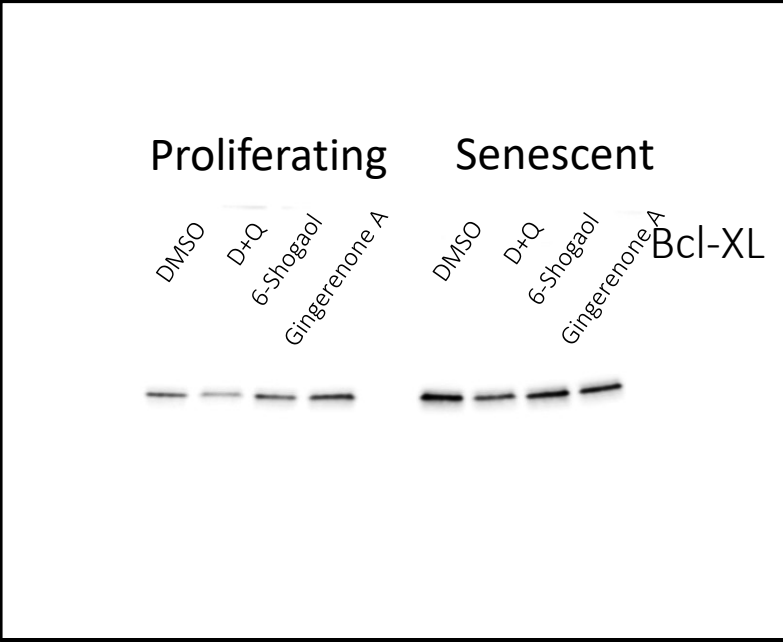

Supplement: S1 Raw images — (PDF) [file pone.0266135.s002.pdf]
